# Genetic testing of children for adult-onset conditions: opinions of the British adult population and implications for clinical practice

**DOI:** 10.1038/ejhg.2014.221

**Published:** 2014-11-05

**Authors:** Shiri Shkedi-Rafid, Angela Fenwick, Sandi Dheensa, Anneke M Lucassen

**Affiliations:** 1 Clinical Ethics and Law at Southampton (CELS), Academic Department of Clinical Genetics, Faculty of Medicine, University of Southampton, Southampton, UK

## Abstract

This study set out to explore the attitudes of a representative sample of the British public towards genetic testing in children to predict disease in the future. We sought opinions about genetic testing for adult-onset conditions for which no prevention/treatment is available during childhood, and about genetic ‘carrier' status to assess future reproductive risks. The study also examined participants' level of agreement with the reasons professional organisations give in favour of deferring such testing. Participants (*n*=2998) completed a specially designed questionnaire, distributed by email. Nearly half of the sample (47%) agreed that parents should be able to test their child for adult-onset conditions, even if there is no treatment or prevention at time of testing. This runs contrary to professional guidance about genetic testing in children. Testing for carrier status was supported by a larger proportion (60%). A child's future ability to decide for her/himself if and when to be tested was the least supported argument in favour of deferring testing.

## Introduction

Professional organisations worldwide are largely unanimous in their recommendation to defer the genetic testing of children for conditions they may develop in the future.^1, 2, 3^ Most suggest that such testing should only take place in childhood if prevention or treatment is available at the time of testing that may alter the future outcome of the condition. Otherwise, testing should be postponed until such time that there is medical benefit or until the child is old enough to make a decision for themselves. Recommendations about genetic testing to see whether someone is a ‘carrier' for a recessive condition or a balanced chromosomal rearrangement are similar: where such carrier status (hereafter called testing for reproductive risks) confers no present or future health problems for the child, but instead possible risks for their children, testing should be postponed in childhood.^1, 2, 4, 5^

Evidence from clinicians in the United Kingdom, the United States and Australia suggests, however, that parents still regularly request testing of their children sometimes years before a medical intervention might be offered.^6, 7^

To the best of our knowledge, only one study has looked at the general public's views on childhood genetic testing for adult-onset conditions, and this was undertaken when genetic testing for future risks was still largely a hypothetical rather than practical possibility. Using a survey distributed to 1000 adults in the United States, in the mid-90s, Wertz and Fletcher^8^ demonstrated that just over half of the respondents (53%) agreed that parents should be able to test their minor children for conditions that were ‘neither preventable nor treatable'.

The field of genetics has progressed considerably in the subsequent two decades, with many more genetic tests now routinely available than when Wertz *et al* undertook their survey. Professional guidance abounds, yet members of the public are those who may ask for testing, live with its consequences or support other family members/friends who have genetic conditions. It is, therefore, important to ascertain their opinions and explore the extent to which they agree/disagree with some of the arguments for deferring genetic testing until adulthood that the guidelines espouse. Better understanding could also improve communication between health care professionals (HCPs) and those referred to genetic services and allow any discrepancies between public expectations and professional guidance to be addressed. Furthermore, as genetic testing is likely to be increasingly ‘mainstreamed' into all clinical specialities,^9^ assessing public expectations is timely.

This paper, which is part of a larger study exploring the views of various stakeholders about childhood genetic testing for adult-onset conditions, reports on the opinions of a representative sample of the adult British population about such testing. It examines the implications of these views for clinical practice in Britain.

## Materials and methods

The study protocol was approved by the University of Southampton, Faculty of Medicine Ethics Committee.

### Participants

An online survey was conducted in April 2013 via YouGov, an independent research company conducting online surveys.^10^ YouGov has a registered panel of over 3.3 million people worldwide, including over 350 000 British adults, chosen on the basis that their sociodemographic characteristics represent the British population. A representative sub-population of the panel members (*n*=3109) was sent an email containing a link to the questionnaire. On completion, participants earned 50 points, which were redeemable for rewards (for example, for 5000 points, panellists can claim a reward of £50 or a variety of other prizes).

### Questionnaire

A structured questionnaire was designed for this study based on current literature and guidelines on childhood genetic testing.^2^ It was reviewed for accuracy, flow and organisation by experts in survey design, genetics, social sciences, philosophy and ethics and by lay and support group representatives. Pilot testing was conducted with 35 individuals of different ages and professional backgrounds, from lay to healthcare professionals. They were asked for their understanding of the questions. While revising the questions, more notice was given to comments made by lay people.

Here we report on respondents' level of agreement/disagreement (strongly agree, agree, neither agree nor disagree, disagree, strongly disagree and don't know) with whether parents should be able to test their children for (1) adult-onset conditions for which there is no current treatment/care and (2) testing for reproductive risks. We also present respondents' views on four arguments in favour of deferring childhood genetic testing (detailed in Table 1), which are utilised in professional guidance. For the full questionnaire, see Supplementary Information.

Sociodemographic data were collected on respondents' gender, age, socioeconomic status and place of residence.

### Data analysis

Analyses were performed using the IBM Statistical Package for the Social Sciences (SPSS) v. 20 (IBM software UK). Descriptive statistics were used to conduct the majority of the analyses. Missing data were excluded from the analysis. Pearson's *χ*^2^-test was used to assess the associations between demographic variables and respondent attitudes. For a parsimonious analysis, the strongly agree and agree responses were grouped together, as well as the strongly disagree and disagree. ‘Neither agree nor disagree' and ‘don't know' responses were also grouped together, hereafter referred to as 'uncertain'. Socioeconomic status categories A, B and C1 were grouped together, as were C2, D and E. Respondents' answers to reasons for deferring testing, listed in Table 1, were ranked so that 0 represents respondents who chose ‘don't know' 1 represents those who thought that the reasons given were not good ones to delay testing; and 2 represents those who thought that they were good reasons to delay testing. Items were then summed to provide each respondent with an overall score (scale score). This score could range from 0, for those who chose ‘don't know' for all four statements, to 8, for those who thought that all four statements were good reasons to delay testing.

For ease of presentation, percentages are rounded to the nearest whole number. A two sided *P*<0.05 was considered statistically significant.

## Results

### Survey respondents

Traditional response rates are not measured in internet surveys.^11^ Instead, an active sampling process is carried out from panellists already signed up to take part in such surveys. Of the 3109 panellists who were approached, 2998 (96%) completed the survey. Participants' demographic characteristics are presented in Table 2.

### Opinions on predictive genetic testing in childhood

#### Testing for adult-onset conditions that the child may develop in the future

**Figure 1:**
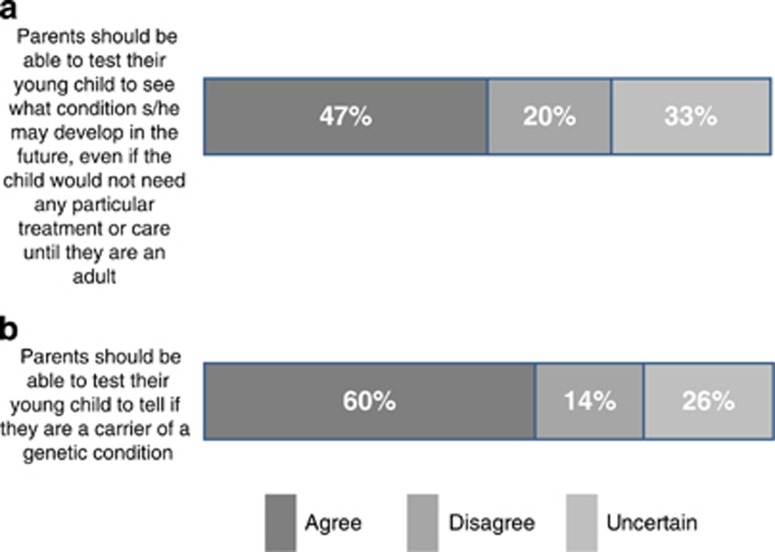
Opinions on testing children for adult-onset conditions with no treatment or care in childhood and testing for reproductive risks. (**a**) Parents should be able to test their young child to see what condition s/he may develop in the future, even if the child would not need any particular treatment or care until they are adults. (**b**) Parents should be able to test their young child to tell if they are a carrier of a genetic condition.

As demonstrated in Figure 1, nearly half of the respondents (47%, *n*=1423) agreed that parents should be allowed to test their children for adult-onset conditions, even if the child would not need any particular treatment or care before adulthood. About a third of the sample (33%, *n*=979) were uncertain about such testing. Only a fifth of the sample (20%, *n*=596) disagreed that parents should be able to test their children for such conditions and it is interesting to note that this opinion is the one most in line with professional guidance.

#### Testing for reproductive risks

The majority of the sample (60%, *n*=1799) agreed that parents should be able to test their child for reproductive risks. About a quarter of the sample (26%, *n*=785) were uncertain. Fourteen percent of respondents (*n*=413) disagreed that parents should be allowed to have such tests, in line with professional guidance.

#### The associations between views about testing and demographic variables

Age was associated with respondents' opinions about the two types of testing, but in different ways. Younger respondents (aged 18–29) were more likely to support testing for adult-onset conditions with no treatment/care in childhood (*P*=0.005). The association between age and opinions about testing for reproductive risks was the other way round, as older respondents (40 and above) were significantly more likely to support such testing in comparison with younger (<40) respondents (*P*<0.001).

Opinions were also significantly associated with respondents' gender. Men were more likely than women to support testing for adult-onset conditions with no treatment/care in childhood (54 *vs* 41%, *P*<0.001) and testing for reproductive risks (64 *vs* 57%, *P*<0.001).

Opinions about testing for adult-onset conditions with no treatment/care in childhood were also associated with socioeconomic status. Respondents in the group category C2DE (broadly manual occupations and ‘working class') were more likely to be uncertain, compared with respondents in category ABC1 (broadly professional occupations and ‘middle class' 36 *vs* 30%, *P*=0.005).

### Opinions on arguments in favour of deferring childhood testing for adult-onset conditions

Table 1 demonstrates respondents' opinions on four arguments in favour of deferring testing for adult-onset conditions.

Removing the child's future ability to make their own decisions was a less supported reason (supported by about a third of respondents). Respondents' opinions on the other three statements were similar, with nearly half opining that these were good reasons to delay testing (45, 45 and 46%, respectively). About a quarter of the sample was uncertain whether or not the four arguments were good reasons to delay testing (25, 24, 25 and 25%, respectively). Looking at respondents' scale scores, 502/2998 respondents (17%) had a score of 0, that is, answered ‘don't know' to all four statements. Twenty-one percent of the sample (637/2998) had a scale score of 8, that is, thought all four arguments were good reasons to delay testing. Respondents with a scale of 4 (569/2998 respondents, 19%) could have thought that all four arguments were not good reasons to delay testing, but could also have given a range of answers for the different statements. The remainder of the sample (43%) had a scale score other than 0, 4 and 8, which would suggest that they gave a range of answers to the various statements.

#### The associations between views about the arguments in favour of deferring testing and demographic variables

Age was significantly associated with respondents' opinions about the four arguments (*P*=0.004, 0.023, 0.008 and 0.000, respectively). Respondents over the age of 60 were more likely to think that removing the child's ability to decide when they are older if they want to be tested (statement 1, Table 1) was not a good reason to delay testing. Respondents aged 25–39 were more likely to think that the fear of stigmatisation/discrimination (statement 2) and potentially misinforming the child about the test results when older (statement 3) were good reasons to delay testing. Younger respondents (aged 18–24) were least likely to think that statement 4 was a good reason to delay testing (that is, testing should only be done when there is medical benefit).

Women were significantly more likely than men to think that all four statements were good reasons to delay testing (*P*<0.001 for all statements).

Socioeconomic status was also significantly associated with respondents' opinions about the four statements, but in different ways. ‘Working class' respondents were more likely to be uncertain about statements 1 and 3 (*P*<0.001 in both cases). ‘Middle class' respondents were more likely to think that the fear of stigmatisation/discrimination and the lack of medical benefit (statements 2 and 4) were good reasons to delay testing (*P*<0.001 in both).

### Associations between respondents' attitudes towards childhood testing for adult-onset conditions and their opinions on arguments in favour of deferring such testing

A correlation was found between respondents' attitudes towards allowing parents to test their children for adult-onset conditions with no current medical utility, and their opinions about each of the various statements. More specifically, those who supported testing were more likely to think that these were not good reasons to defer testing (60, 51, 48 and 50%, respectively, in the order detailed in Table 1, *P*<0.001); those who objected to testing were more likely to agree that these were good reasons to delay testing (70, 78, 74 and 82%, respectively, *P*<0.001); and those who were uncertain about testing were most likely to remain uncertain about arguments in favour of delaying testing (49, 46, 46 and 46%, respectively, *P*<0.001).

## Discussion

Our survey was designed to gain an insight into the reactions of a representative sample of the adult British population to the issue of genetic testing in children for adult-onset conditions and reproductive risks.

Our findings show that 47% of the sample agreed that parents should be able to test their children for adult-onset conditions with no treatment/care at the time of testing, even if (at least some of them) agreed that there are good reasons to delay such testing. This finding is consistent with our current clinical experience and national professional discussions such as the Genethics forum,^6^ where clinicians report that parents regularly request such testing and that they are unsure how to deal with these in the face of professional guidance.

Testing for reproductive risks was supported by 60% of the sample, representing a greater proportion than those who supported predictive testing for conditions that the child herself may develop in the future. We find this difference interesting as, in both cases, there is no medical benefit to knowing the result before the child can make up their own mind if and when to be tested. Interestingly, in its response to the 1994 report of the UK Clinical Genetics Society on childhood genetic testing, the Genetic Interest Group (now Genetic Alliance UK), an umbrella organisation for patient organisations supporting individuals affected by genetic conditions in the United Kingdom, distinguished testing for reproductive risks and testing for adult-onset conditions.^12^ Testing for reproductive risks was considered ‘less serious',^12^ with potential benefits (for example, gradual adaptation to the information).^12, 13^ Our findings suggest that participants saw knowledge as beneficial in and of itself.

There are a number of potential explanations why nearly half of our respondents supported genetic testing for adult-onset conditions and over half supported testing for reproductive risks. One possible explanation is that, although there are no known interventions, people may believe that parents can do something with the information, such as change their child's diet, be alert to new interventions, prepare their children and themselves for symptom onset, encourage children who are carriers to adopt various lifestyle practices and inform them of their possibly increased reproductive risks before they have children.^13, 14, 15^

It could also be that the big drive for personalised medicine and media coverage of the achievements in the field of genetics have resulted in the general impression that genetic testing is mainly beneficial, with few downsides or limitations.^16^ At a time when genetic/genomic testing is becoming cheaper and more accessible in both healthcare and direct-to-consumer settings, people may not appreciate that the technical ease of obtaining a result will not always reflect the difficulties in interpreting and incorporating the results into daily life, nor that there may be disadvantages to certain types of testing. It has been previously demonstrated that people often do not fully appreciate the complexity of the consequences of genetic testing until they receive their results.^17, 18^

Another potential explanation of respondents' general support of childhood testing is that they support parental ‘rights' to decide whether or not to test their children, even if they recognise that there are good reasons to delay testing.^13, 19, 20, 21^ This may also explain why the ability of the future child to decide for themselves if and when to be tested was the least supported argument for deferring testing (Table 1). Previous research has showed that healthy adults who were tested in childhood for X-linked and autosomal-recessive conditions, or balanced chromosomal rearrangements, largely felt that the decision to test a child should be made by the parents.^22, 23, 24^ Yet, the importance that is attributed by HCPs to the preservation of future decision-making ability has been repeatedly reiterated in empirical studies looking at HCPs' views on childhood testing.^7, 8, 25^ The inclination to protect future autonomy may explain why HCPs are more willing to approve testing for adult-onset conditions in older minors as opposed to younger ones, even if there are still many years before the predicted onset of the condition in question.^7^

Whereas HCPs focus on medical benefit to the child, public concepts of children's interests may be much broader, which may make it harder for HCPs to convey the potential benefit of allowing the child to decide for themselves in the future if and when to be tested for conditions with no treatment/care in childhood. Our findings suggest that people may relate more easily to other reasons to defer testing, such as the fear of discrimination and stigmatisation, the lack of medical utility and the possibility of misinforming the child in the future about their test results.

With the likely ‘mainstreaming' of genetics, it might be that (in the UK as well as in other countries) HCPs outside the specialty of clinical genetics will discuss childhood genetic testing with parents with possibly less time for detailed discussions. Media coverage of highly unusual cases (for example, http://www.bbc.co.uk/news/uk-england-stoke-staffordshire-27832039) might also prompt parents to request testing for their children at younger ages. The potential gap between parents' and HCPs' understandings of what genetic testing will tell them about their children's futures may, therefore, be difficult to bridge. We suggest that wider public engagement exercises are designed that may help towards a greater concordance between public and professional views.

This study has some limitations: we did not collect data on the parental status of respondents, offspring ages and respondents' prior knowledge of genetics. Although it might have been interesting to see if parental status influenced people's views about predictive genetic testing in childhood, this was not what we set out to do. We cannot rule out the possibility that better understanding of various aspects of testing and parental status would influence opinions. However, a large proportion of respondents did not have clear opinions about the various types of testing, which is likely to reflect a lack of prior knowledge about the issues.

As this survey was used to provide general trends and associations, categories with multiple options (such as Likert scale with more than three options and demographics with more than two to three groups) were grouped together (such as degree of agreement/disagreement, socioeconomic status). A more detailed analysis might have better identified subgroups within the respondents that were more likely to request childhood testing, and disagree with the reasons brought forward by professional guidelines to delay testing. For most of the respondents, we expect these questions were hypothetical, in that they would not actually have sought genetic testing of their children. Previous research, mainly on predictive testing for Huntington's disease, demonstrated that such hypothetical decisions do not necessarily represent actual behaviour.^26, 27^ Nevertheless, even if our results do not represent the actual decisions parents would make regarding testing of their children, they give an indication about how they might feel, at least initially, were the situation to become a reality for them. This information could be incorporated into consultations by HCPs.

With regards to respondents' answers to the four statements in favour of deferring testing (Table 1), it could be that at least some of the respondents chose the same answer (good reason/not good reason/don't know) for all statements. These participants might have been overall in favour or not in favour of childhood testing and answered accordingly, regardless of the reason provided. Alternatively, participants might not have thought carefully about the differences between the statements. However, at least 43% of participants gave different answers for each reason. Another interesting question, beyond the scope of the current study, is whether respondents' views would have been different if they were asked about testing minors for severe and untreatable adult-onset conditions, especially if they were provided with information about the uptake of such tests by at-risk adults.

In summary, our study shows that the British adult public is more likely to either be uncertain or in support of childhood testing for adult-onset conditions for which there is no intervention at the time of testing. Testing to assess future reproductive risks is also supported. Only a minority of participants agreed with HCPs' attitudes and professional guidelines about delaying such testing until it is medically actionable, or until the child is old enough to make a decision for themselves. Whereas HCPs think that preservation of future autonomy is an important reason to defer testing, our respondents were more likely to think that this was not a good reason, or to remain uncertain.

## Figures and Tables

**Table 1:** Opinions on arguments in favour of deferring testing for adult-onset conditions

*Are these good reasons to delay testing?*	*A good reason*	*Not a good reason*	*Not sure*
It removes the child's ability to decide when they are older if they want to be tested or not	37%, *n*=1103	38%, *n*=1129	25%, *n*=765
The result may make the child feel stigmatised or discriminated against as they grow up	45%, *n*=1342	31%, *n*=925	24%, *n*=731
The child may be misinformed about the condition they might develop if they are not involved in the decision about testing	45%, *n*=1343	30%, *n*=893	25%, *n*=762
There is no medical benefit to testing now; the test should only be done when there is benefit	46%, *n*=1366	29%, *n*=886	25%, *n*=746

**Table 2:** Characteristics of participants

*Participants,* n *(%)*	*Characteristic*
*Age*	
363 (12)	18–24
764 (25)	25–39
1025 (34)	40–59
845 (28)	>60
	
*Gender*	
1541 (51)	Female
1457 (49)	Male
	
*Social grade*^a^	
1709 (57)	ABC1
1289 (43)	C2DE
	
*Place of residence*	
384 (13)	London
738 (25)	Rest of South
642 (21)	Midlands/Wales
974 (32)	North
261 (9)	Scotland

a The socioeconomic classification used by YouGov is derived from the National Readership Survey (NRS): ABC1 represents people who are employed in professional occupations (broadly ‘middle class'); C2DE represents manual workers and those on subsistence levels of income who may or may not be employed (broadly ‘working class').
