# Supplementary material for: Genetic testing of children for adult-onset conditions: opinions of the British adult population and implications for clinical practice
Source: Eur J Hum Genet. 2014 Nov 5;23(10):1281–5. doi: 10.1038/ejhg.2014.221 (PMC4592073; doi:10.1038/ejhg.2014.221)
Supplement: Supplementary Information [file ejhg2014221x1.doc]

**QUESTIONNAIRE:**

A genetic test is sometimes used to diagnose whether a child has an inherited condition if they have particular signs or symptoms. Other genetic tests can predict a condition or disease which a child may develop in the future.

1. Thinking about genetic tests that predict future conditions (those that won’t appear for many years from now) and which say nothing about the child’s current health, please state your opinion about the following statements:

1. Parents should be able to test their young child to see what condition s/he may develop in the future, even if the child would not need any particular treatment or care until they are an adult.

□ Strongly agree

□ Agree

□ Disagree

□ Strongly disagree

□ Neither agree nor disagree

□ Don’t know

1. Parents should only be able to test their young child to see what condition s/he may develop in the future if treatment or care is available now.

□ Strongly agree

□ Agree

□ Disagree

□ Strongly disagree

□ Neither agree nor disagree

□ Don’t know

2. Some people think that parents should not be allowed to test their young child for conditions that they may develop in adulthood. Below are some of the reasons that are given for this.

Thinking about these, please state whether you think they are or are not good enough reasons to delay testing.

1. It removes the child's ability to decide when they are older if they want to be

tested or not.

<1> This is a good reason to delay testing

<2> This is not a good reason to delay testing

<3> Don’t know

1. The result may make the child feel stigmatised or discriminated against as they

grow up.

<1> This is a good reason to delay testing

<2> This is not a good reason to delay testing

<3> Don’t know

1. The child may be misinformed about the condition they might develop if they are not involved in the decision about testing.

<1> This is a good reason to delay testing

<2> This is not a good reason to delay testing

<3> Don’t know

1. There is no medical benefit to testing now; the test should only be done when

there is benefit.

<1> This is a good reason to delay testing

<2> This is not a good reason to delay testing

<3> Don’t know

3. Some types of genetic tests do not test whether someone will be affected by a condition, but instead they can tell whether that person is a “carrier” for a particular condition. Being a “carrier” means the individual will not develop the condition themselves, but they might have children who have the condition. Please state how much you agree or disagree with the following statement:

Parents should be able to test their young child to tell if they are a carrier of a genetic condition.

□ Strongly agree

□ Agree

□ Disagree

□ Strongly disagree

□ Neither agree nor disagree

□ Don’t know
